# A Pan-Cancer Analysis of the Oncogenic and Immunogenic Role of m6Am Methyltransferase PCIF1

**DOI:** 10.3389/fonc.2021.753393

**Published:** 2021-11-23

**Authors:** Ming-Zhu Jin, Yi-Gan Zhang, Wei-Lin Jin, Xi-Peng Wang

**Affiliations:** ^1^ Department of Obstetrics and Gynecology, Xinhua Hospital Affiliated to Shanghai Jiao Tong University School of Medicine, Shanghai, China; ^2^ Institute of Cancer Neuroscience, Medical Frontier Innovation Research Center, The First Hospital of Lanzhou University, The First Clinical Medical College of Lanzhou University, Lanzhou, China

**Keywords:** PCIF1, N6,2′-O-dimethyladenosine (m6Am), pan-cancer analysis, immune checkpoint genes (ICGs), posttranslational modification

## Abstract

**Background:**

Phosphorylated CTD-interacting factor 1 (PCIF1) is identified as the only known methyltransferase of N6,2′-O-dimethyladenosine (m6Am) in mRNA. However, its oncogenic and immunogenic role in cancer research is at an initial stage.

**Methods:**

Herein, we carried out a pan-cancer analysis of PCIF1, with a series of datasets (e.g., TIMER2.0, GEPIA2, cBioPortal).

**Results:**

PCIF1 expression was higher in most cancers than normal tissues and was discrepant across pathological stages. Highly expressed PCIF1 was positively correlated with overall survival (OS) or disease-free survival (DFS) of some tumors. PCIF1 expression had a positive correlation with CD4^+^ T-cell infiltration in kidney renal clear cell carcinoma (KIRC), CD8^+^ T cells, macrophages, and B cells in thyroid carcinoma (THCA), and immune checkpoint genes (ICGs) in LIHC but a negative correlation with CD4^+^ T cells, neutrophils, myeloid dendritic cells, and ICGs in THCA. It also affected tumor mutational burden (TMB) and microsatellite instability (MSI) of most tumors.

**Conclusion:**

PCIF1 expression was correlated with cancer prognosis and immune infiltration, suggesting it to be a potential target for cancer therapy.

## Introduction

Epitranscriptomics has been an attractive field of recent cancer researches. Posttranslational modification (PTM) of mRNA includes N4-acetylcytidine (ac4C), N1-methyladenosine(m1A), N6-methyladenosine (m6A), 5-methylcytosine (m5C), 5-hydroxymethylcytidine (hm5C), N6,2′-O-dimethyladenosine (m6Am), 7-methylguanosine (m7G), inosine (I), and pseudouridine (Ψ). Among which, m6A is one of the most studied PTM in cancer researches. Accumulating evidence has suggested the participation of other mRNA modification in oncogenesis or cancer development. Zhang et al., for example, have found NAT10-mediated mRNA ac4C modification in promotion of gastric cancer (GC) metastasis and epithelial-to-mesenchymal transition (EMT) (1). The Y box binding protein 1 (YBX1)-mediated m5C modification promoted mRNA stability, which activated oncogenesis of urothelial carcinoma of the bladder (2). The m6Am was another established reversible RNA modification, conducive to mRNA translation, expression, and stability. Previous studies have identified phosphorylated CTD-interacting factor 1 (PCIF1) as the only known m6Am writer protein (3–7) and fat mass and obesity-associated protein (FTO) as an eraser protein for mRNA (7–9). METTL4 was a m6Am writer protein for U2 small nuclear RNA (snRNA), which regulated RNA splicing (10). PCIF1 was involved in glucose homeostasis through regulating the function or survival of pancreatic beta cell (11) and viral infectivity (12). PCIF1-mediated m6Am RNA modification was associated with reduced mice weight (13). Recently, Relier and colleagues have shown that inhibition of nuclear PCIF1-mediated m6Am modification in mRNA led to reduced colorectal cancer stem cell abilities, which was the first and the only study so far that revealed the role of m6Am of mRNA in cancer (14). However, biological and functional role of m6Am in cancer research remains obscure. We attempt to primarily explore the oncogenic and immunogenic role of PCIF1 on the basis of the pan-cancer analysis.

## Materials and Methods

### Expression Analysis

We obtained expression difference of PCIF1 between tumor and normal tissues based on Cancer Genome Atlas (TCGA) database. For those tumors that lack data for normal tissues, we used genotype-tissue expression (GTEx) database as a **Supplementary Material**. We also obtained violin plots of the PCIF1 expression difference in different pathological stages of TCGA tumors *via* the “Pathological Stage Plot” module of GEPIA2 (http://gepia2.cancer-pku.cn/) (15). The log2 (transcripts per million (TPM) +1) were applied as log-scale for violin plots. We also examined specificity of PCIF1 RNA expression in different tissues, blood cell lineages, and single cell types *via* the consensus dataset composed of Human Protein Atlas (HPA), GTEx, and FANTOM5 datasets on the Human Protein Atlas website (https://www.proteinatlas.org/). We applied *p*-value as the measurable indicator for statistical hypothesis during the whole study and suggested a significant difference for a result of *p* < 0.01, a statistical difference for a result of *p* < 0.05, and no statistical difference for *p* > 0.05 herein and in the rest of the content (16).

### Survival Prognosis Analysis

We evaluated the correlation of PCIF1 RNA expression and overall survival (OS), disease-free survival (DFS/RFS) *via* “Survival Analysis” module of GEPIA2 (15). Cutoff-high (50%) and cutoff-low (50%) values were applied as the expression thresholds.

### Genetic Alteration Analysis

We utilized the cBioPortal web (https://www.cbioportal.org/) to explore genetic alteration characteristics of SND1 by pressing “quick search” and entering PCIF1 (17, 18). The expected results including a three-dimensional structure diagram of PCIF1, alteration frequency, mutation type, copy number alteration (CNA), and PTM sites *via* “Cancer Types Summary”, “Plots”, and “Mutations” module. We also obtained the differences of OS, DFS, progression-free survival (PFS), and disease-specific survival (DSS) for pan-cancer with or without PCIF1 alteration *via* the “Comparison/Survival” module and generated Kaplan-Meier plots with log-rank *p*-value.

### Phosphorylation Analysis

The protein expression and phosphoprotein level (S30, S116, S140, S144, S159, T150, T158) of PCIF1 was obtained from the UALCAN website (http://ualcan.path.uab.edu/analysis-prot.html) on the basis of clinical proteomic tumor analysis consortium (CPTAC) dataset (19). In UALCAN, only data for six tumors were available including ovarian cancer, colon cancer, clear-cell renal cell carcinoma (RCC), uterine corpus endometrial carcinoma (UCEC), breast cancer, and lung adenocarcinoma (LUAD).

### Immune Infiltration Analysis

The TIMER (http://timer.cistrome.org), CIBERSORT (https://cibersort.stanford.edu/), EPIC (https://gfellerlab.shinyapps.io/EPIC_1-1), MCP-Counter (http://github.com/ebecht/MCPcounter), quanTIseq (http://icbi.i-med.ac.at/software/quantiseq/doc/index.html), and xCell (http://xcell.ucsf.edu/) were applied for evaluation of PCIF1 immune infiltration in 33 TCGA tumors. The purity and infiltration level of CD8^+^, CD4^+^, neutrophil, macrophage, and B cell with log2 TPM as scale were illustrated by the “Immune Estimation” module of TIMER2.0. The Rho and *p*-values were obtained from Spearman’s correlation test.

### Immune Checkpoint Genes, Tumor Mutational Burden, and Microsatellite Instability analysis

We tested the correlation of PCIF1 expression and eight immune checkpoint genes (ICGs) including CD274, CTLA4, HAVCR2, LAG3, PDCD1, PDCD1LG2, SIGLEC15, and TIGIT.

Additionally, we provided Spearman’s correlation analysis of tumor mutational burden (TMB), microsatellite instability (MSI), and PCIF1 expression. The data were acquired from TCGA database, downloaded from the Genomic Data Commons (GDC) data portal website (https://portal.gdc.cancer.gov/) and managed with R software v4.0.3 for statistical analysis. For reliable immune score evaluation, we use immunedeconv, an R package that integrates six latest algorithms, including TIMER, CIBERSORT, EPIC, MCP-counter, quanTIseq, and xCell (https://grst.github.io/immunedeconv) (20).

### PCIF1-Related Gene Enrichment Analysis

To figure out interplays of PCIF1 protein, we carried out a series of gene-enrichment analysis. We first obtained top 100 PCIF1-binding proteins in *Homo sapiens via* the STRING database (http://string-db.org). Parameters we used were “evidence” for meaning of network edges (“evidence”), “low confluence (0.150)” for minimum required interaction score, “100” for max interactors, and no limitations/”experiment” for active interaction sources to get predicted functional partners of PCIF1. We used “Similar Gene Detection” module of GEPIA2 get top 100 PCIF1-correlated genes. Top 5 correlated genes were selected for further analysis. We applied the “Correlation Analysis” module of GEPIA2 to Pearson’s test of the top 5 PCIF1-correlated genes. Venn diagram was provided to obtain an intersection of 100 PCIF1 correlated genes from GEPIA2 and 100 interacted proteins from STRING with Evenn (http://www.ehbio.com/test/venn/). We also conducted global percentage and relation network diagram to depict pathway activity of PCIF1, its interacted proteins, and correlated genes *via* GSCALite, a powerful and widely applied tool for analyzing cancer multiomics and drug sensitivity (http://bioinfo.life.hust.edu.cn/web/GSCALite/) (21).

## Results

### PCIF1 Expression Analysis

**Figure 1:**
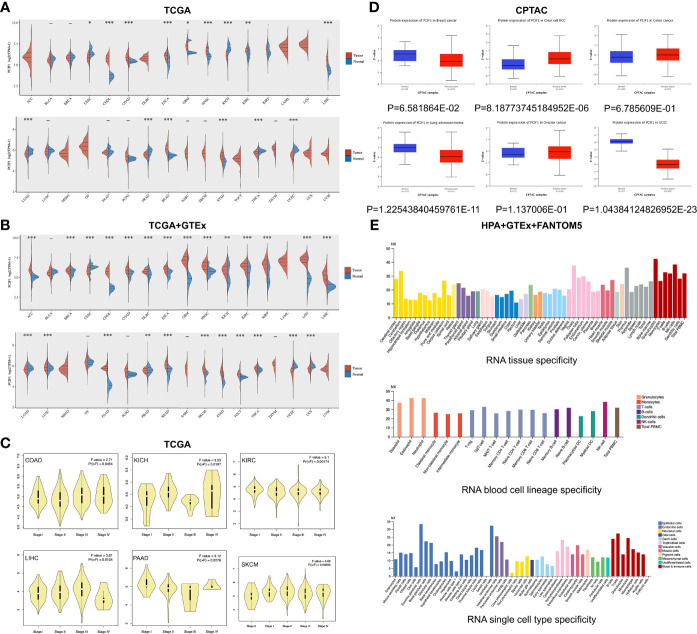
Differential expression of PCIF1. **(A)** Expression of *PCIF1* gene in different TCGA tumors. ^*^ *p* < 0.05; ^**^ *p* < 0.01; ^***^ *p* < 0.001. **(B)** For ACC, DLBC, LGG, MESO, OV, SKCM, and UCS, data of PCIF1 expression in normal tissues were obtained from GTEx databases as **Supplementary Materials**. ^**^ *p* < 0.01; ^***^ *p* < 0.001. **(C)** *PCIF1* gene expressions of different pathological stages in COAD, KICH, KIRC, LIHC, PAAD, and SKCM. **(D)** Expression of PCIF1 total protein of breast cancer, clear-cell RCC, colon cancer, lung adenocarcinoma, ovarian cancer, and UCEC, collected from the CPTAC dataset. **(E)** PCIF1 RNA expression in different tissue, blood cell lineage, and single cell type based on consensus datasets (HPA+GTEx+FANTOM5).

In our study, we first assessed differential expression of PCIF1 among tumor and adjacent normal tissues in 28 types of tumors based on data collected from the TCGA database and GTEx database as **Supplementary Materials**. Expression of PCIF1 had a significant difference in 25 of 28 tumor types like adrenocortical carcinoma (ACC), breast invasive carcinoma (BRCA), colon adenocarcinoma (COAD), glioblastoma multiforme (GBM), kidney chromophobe (KICH), and prostate adenocarcinoma (PRAD) (*p* < 0.01), while no statistic difference in the rest of tumor types (*p* > 0.05) **(** **Figures 1A, B**). Violin plots showed that the difference expression of PCIF1 had statistical significance in each pathological stages of COAD, KICH, kidney renal clear cell carcinoma (KIRC), liver hepatocellular carcinoma (LIHC), pancreatic adenocarcinoma (PAAD), and skin cutaneous melanoma (SKCM) (**Figure 1C**). Additionally, we conducted total protein expression distribution of the PCIF1 among primary tumor and normal tissues from clinical proteomic tumor analysis consortium (CPTAC) dataset. Six available tumors were chosen including breast cancer, clear cell renal cell carcinoma (RCC), colon cancer, ovarian cancer (OV), lung adenocarcinoma (LUAD), and uterine corpus endometrial carcinoma (UCEC). The PCIF1 protein expression in UCEC and LUAD was much more higher in normal tissue than primary tumor (*p* < 0.01) on the contrary to that in clear cell RCC (*p* < 0.01). There was no significant difference in the PCIF1 protein expression between normal and tumor tissues in the rest of the three cancers including breast cancer, colon cancer, and OV (*p* > 0.05) (**Figure 1D**). PCIF1 RNA specificity was low in different tissues, blood cell lineages, single cell types (**Figure 1E**), and cell lines, which was not enclosed.

### PCIF1 Survival Prognosis Analysis

**Figure 2:**
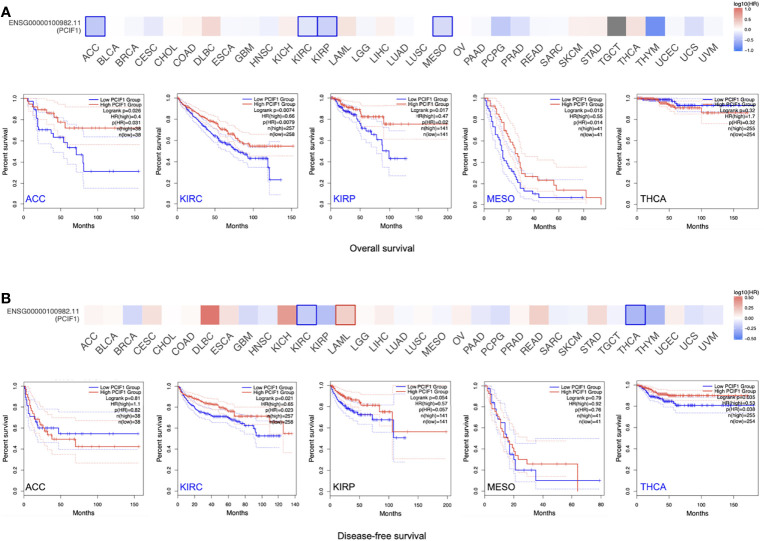
Correlation of PCIF1 gene expression and overall survival, disease-free survival of TCGA tumors. **(A)** Correlation of *PCIF1* gene expression in ACC (*p* < 0.05), KIRC (*p* < 0.05), KIRP (*p* < 0.05), MESO (*p* < 0.05), and THCA (*p* > 0.05) and overall survival of corresponding tumors. **(B)** Correlation of *PCIF1* gene expression in ACC (*p* > 0.05), KIRC (*p* < 0.05), KIRP (*p* > 0.05), MESO (*p* > 0.05), and THCA (*p* > 0.05) and disease-free survival of corresponding tumors.

The OS and RFS significance map of those who had statistical difference, namely, ACC, KIRC, kidney renal papillary cell carcinoma (KIRP), mesothelioma (MESO), SKCM, and thyroid carcinoma (THCA) were demonstrated (**Figure 2**). As shown in **Figure 2A**, the high expression of PCIF1 was correlated with higher OS of ACC (*p* = 0.031), KIRC (*p* = 0.0079), KIRP (*p* = 0.02), and MESO (*p* = 0.014), while only high expression of PCIF1 was correlated with a better RFS (*p* = 0.023) in KIRC. Highly expressed PCIF1 was positively linked with RFS of THCA (*p* = 0.038) but had no statistic difference in OS of THCA (*p* > 0.05). Additionally, we have analyzed the correlation of PCIF1 expression and PFS and DSS data on TCGA datasets (**Supplementary Figure S1**). Interestingly, there is a correlation between highly expressed PCIF1 and poorer DSS of cervical squamous cell carcinoma and endocervical adenocarcinoma (CESC) (*p* = 0.0012) and COAD (*p* = 0.0482). The above data suggested that PCIF1 expression was differentially associated with the prognosis of specific tumor types and mostly presented a positive correlation with OS and RFS.

### PCIF1 Genetic Alteration Analysis

**Figure 3:**
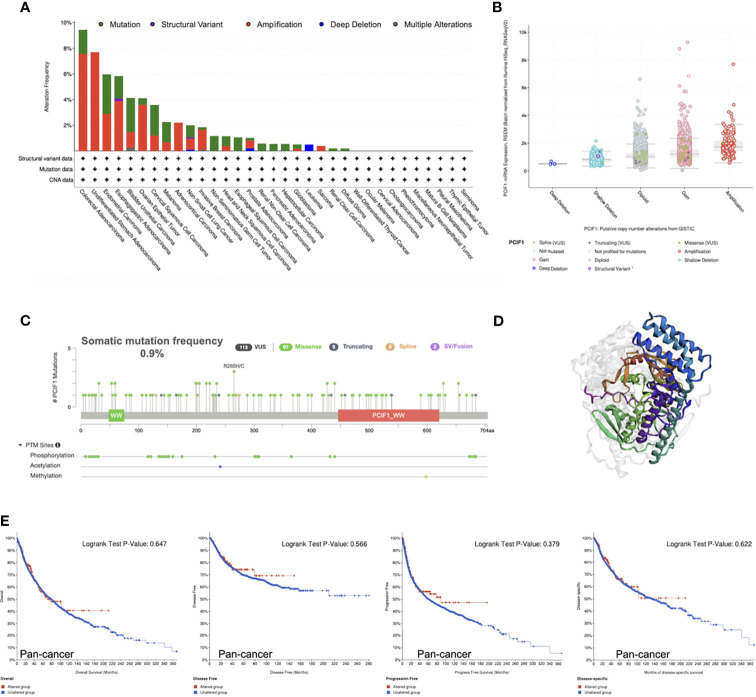
Mutation characteristics of PCIF1 obtained from the cBioPortal website. **(A)** Mutation type and frequency of PCIF1. **(B)** Putative copy-number alterations of PCIF1. **(C)** Mutation frequency and posttranslational modification sites of PCIF1. **(D)** Three-dimensional structure of PCIF1. **(E)** Correlation of PCIF1 mutation and overall, disease-free, progression-free, and disease-specific survival in pan-cancer.

Furthermore, genetic alteration status of PCIF1 were tested and shown (**Figure 3**). COAD possessed the highest gene alteration frequency of PCIF1 (9.43%), with 11 of 594 (1.85%) “mutation” cases and 45 of 594 (7.58%) “amplification” cases. The highest mutation frequency (3.07%) of PCIF1 appeared in endometrial carcinoma. There was a fairly low frequency of structural variant in esophagogastric adenocarcinoma, nonsmall-cell lung cancer (NSCLC) and deep deletion in leukemia, NSCLC, and prostate adenocarcinoma (**Figures 3A, B**). The total somatic mutation frequency was 0.9%, in which missense mutations were in the majority. R265H/C was detected in two cases of UCEC and one case of COAD (**Figure 3C**), while the role of amino acid motifs remained unknown. However, altered PCIF1 had no correlation with OS, RFS, PFS, and DSS of pan-cancer and each tumor type (*p* > 0.05) (**Figure 3E**).

### PCIF1 Protein Phosphorylation Analysis

**Figure 4:**
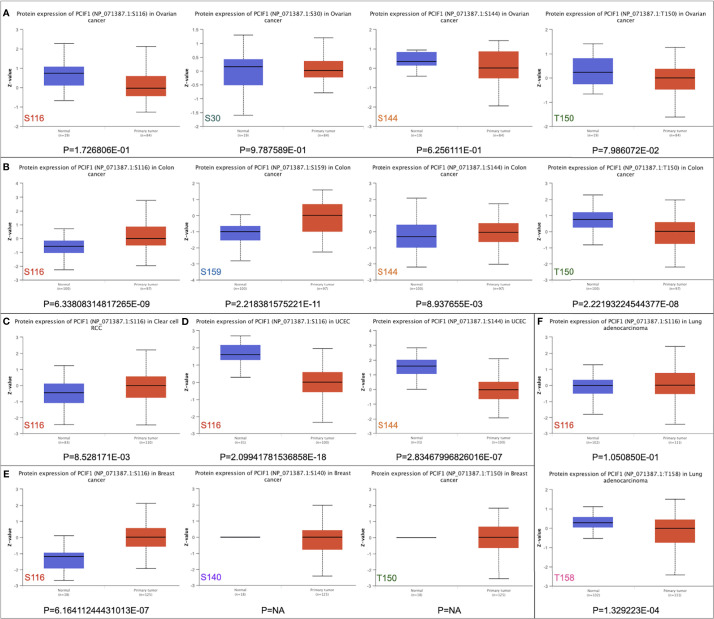
Phosphorylation of PCIF1 protein between normal and primary tumor sites based on the CPTAC dataset. **(A)** Ovarian cancer. **(B)** Colon cancer. **(C)** Clear-cell RCC. **(D)** UCEC. **(E)** Breast cancer. **(F)** Lung adenocarcinoma.

We also analyzed differences in phosphorylation of PCIF1 among normal tissues and tumor lesions (OV, colon cancer, clear cell RCC, UCEC, breast cancer, and lung adenocarcinoma) based on CPTAC dataset (**Figure 4**). PCIF1 phosphorylation level in OV had no significant difference among normal tissues and tumor sites (**Figure 4A**, *p* > 0.05). The S116, S159, and S144 loci of PCIF1 possessed a higher phosphorylation level in colon cancer tissues, but the phosphorylation level at the T150 locus was higher in normal tissues with statistical difference (**Figure 4B**, *p* < 0.05). An increased phosphorylation level was also seen in clear cell RCC and breast cancer tissues, with statistical difference, compared with normal tissues (**Figures 4C, E**, *p* < 0.05) while the situation was opposite in UCEC (**Figure 4D**, *p* < 0.05). PCIF1 phosphorylation of the T158 locus was elevated in normal tissues compared with lung adenocarcinoma tissues; PCIF1 phosphorylation of the S116 locus had no statistical difference between the two groups. PCIF1 was predicted to be located intracellularly (nucleoplasm, microtubules, cytokinetic bridge). Whether the phosphorylation of PCIF1 has an effect on its location or its function (e.g., catalytic activity) remains unknown, requiring more investigations.

### PCIF1 Immune Infiltration Analysis

**Figure 5:**
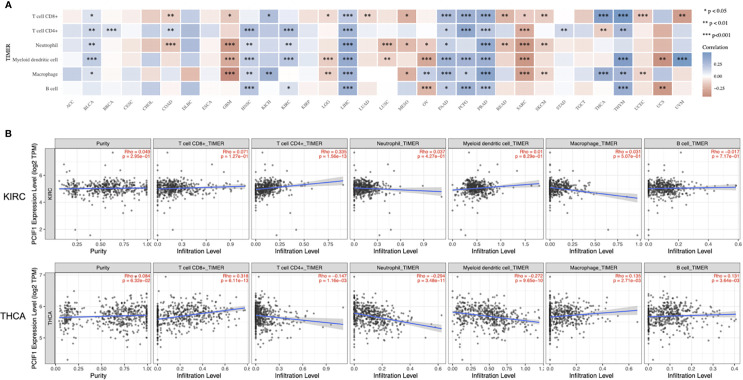
Correlation between PCIF1 and immune infiltration. **(A)** Correlation between PCIF1 and immune infiltration of CD8^+^ T cell, CD4^+^ T cell, neutrophil, myeloid dendritic cell, macrophage, and B cell. **(B)** Correlation between PCIF1 and immune infiltration in KIRC and THCA. *p < 0.05; **p < 0.01; ***p < 0.001.

We applied a series of analytical tool on TIMER2.0 including TIMER, CIBERSORT, EPIC, MCP-counter, quanTIseq, and xCell to investigate immune infiltration of PCIF1 (**Figure 5** and **Supplementary Figure S2**). **Figure 5** displays the expression level of PCIF1 in CD8+ T cell, CD4+ T cell, neutrophil, myeloid dendritic cell (MDC), macrophage, and B cell in 33 tumor types, among which PCIF1 expression harbored a significant difference in immune cells in liver hepatocellular carcinoma (LIHC) and prostate adenocarcinoma (PRAD). The xCell had revealed that the significant difference of PCIF1 expression in monocytes and macrophages (“macrophage,” “macrophage M1,” and “macrophage M2”) of GBM and sarcoma (SARC). Furthermore, we created linear regressions in ACC, KIRC, KIRP, and THCA to see how two variables fit. The selected tumors are those who had statistical difference not only in expression among normal tissue and tumor sites but also in OS or RFS (**Figure 5** and **Supplementary Figure S3**). Six immune cell types were chosen according to xCell. **Figure 5** only included KIRC and THCA, since there was no significant difference of PCIF1 immune infiltration in ACC and KIRP (*p* > 0.05). PCIF1 expression in CD4+ T cell, neutrophil, MDC and B cell of KIRC, and CD8+ T cell and macrophage of THCA was positively correlated with their infiltration level, while negatively correlated in CD4+ T cell of THCA.

### PCIF1 Expression and Immune Checkpoint Genes

**Figure 6:**
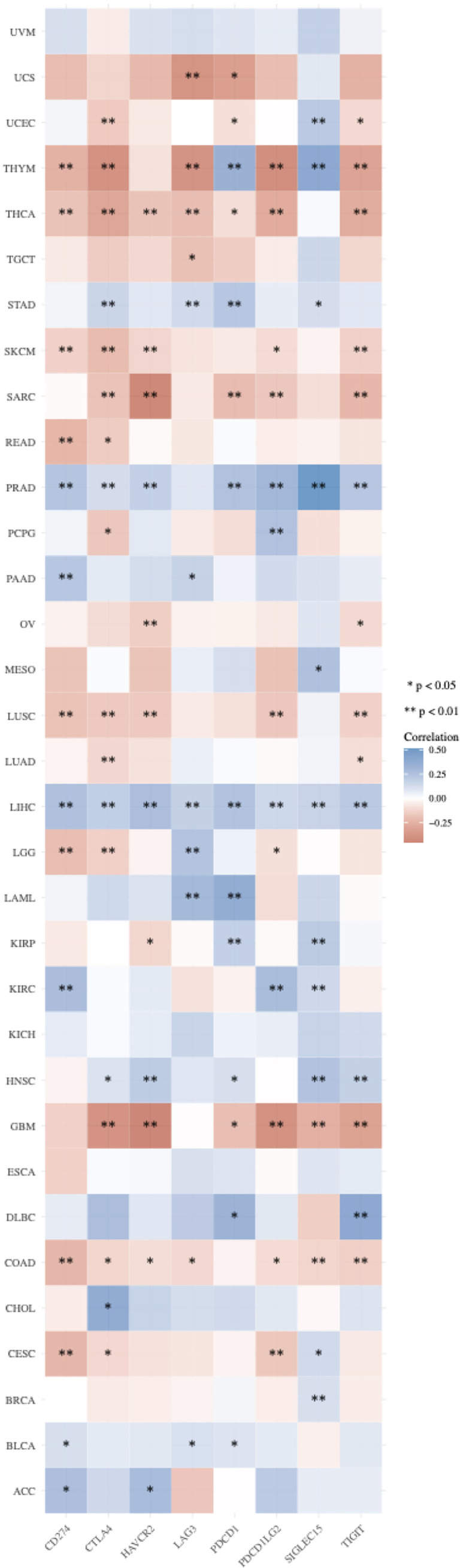
Correlation between PCIF1 and immune checkpoint genes in TCGA tumors. ^*^ *p* < 0.05; ^**^ *p* < 0.01.

Immune microenvironment influences therapeutic response of immune checkpoint blockade. Expression profile of ICGs is linked with immune infiltration, response of immunotherapy, and prognosis. Therefore, we analyzed eight classical ICGs including CD274 (PD-L1), CTLA4 (CD152), HAVCR2 (TIM3), LAG3, PDCD1 (PD-1), PDCD1LG2, SIGLEC15, and TIGIT that have an either positive or negative correlations with PCIF1 expression in all 33 TCGA tumors (22) (**Figure 6**). **Figure 6** suggests that PCIF1 expression had a strong correlation with almost all the selected immune checkpoint genes in LIHC, thymoma (THYM), THCA, PRAD, and COAD, consistent with data of PCIF1 immune infiltration. No relationships were found in uveal melanoma (UVM), KICH, and esophageal carcinoma (ESCA).

### PCIF1 Expression and TMB or MSI

**Figure 7:**
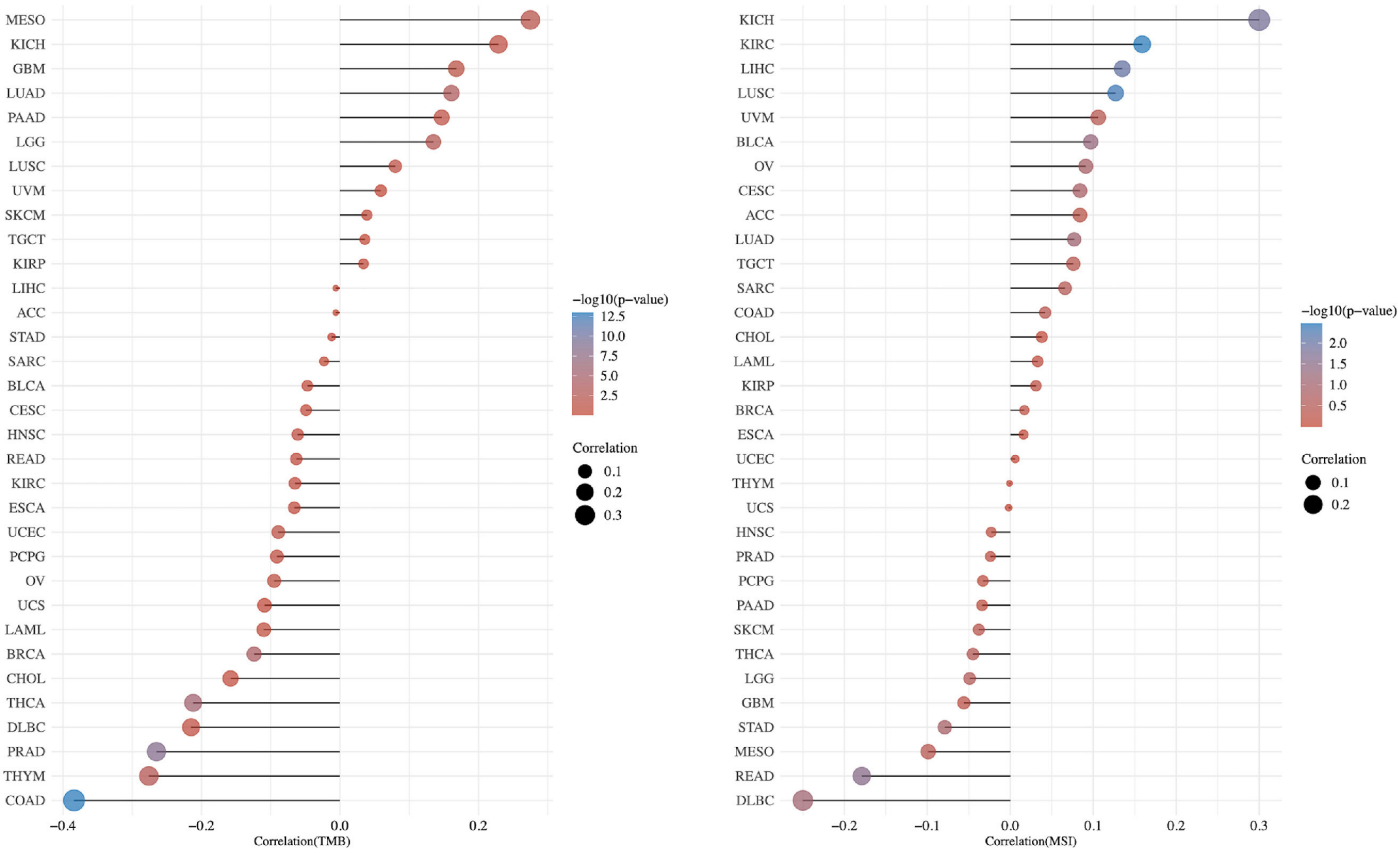
Correlation between PCIF1 and TMB and MSI in TCGA tumors.

As TMB (23) and MSI (24) are considered biomarkers for immunotherapy, we also depicted the correlation between PCIF1 expression and TMB or MSI with bubble diagram to further evaluate its immunogenicity (**Figure 7**). The expression of PCIF1 was positively correlated with TMB of MESO, GBM, LUAD, and brain lower-grade glioma (LGG) but negatively correlated with TMB of COAD, THYM, PRAD, THCA, BRCA, and UCEC (*p* < 0.05). The expression of PCIF1 was positively correlated with MSI of KICH, KIRC, LIHC, and lung squamous cell carcinoma (LUSC) but negatively correlated with MSI of rectum adenocarcinoma (READ) (*p* < 0.05). Interestingly, there were no overlaps between TMB and MSI groups.

### PCIF1-Related Gene Enrichment Analysis

**Figure 8:**
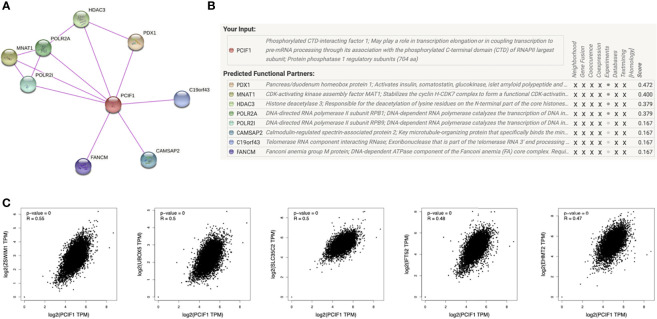
PCIF1-related gene enrichment analysis. **(A, B)** Experimentally interacted proteins of PCIF1 obtained by STRING. **(C)** Correlation between PCIF1 and top 5 PCIF1-correlated genes (ZSWIM1, UBOX5, SLC35C2, IFT52, RHMT2) interacted proteins obtained by GEPIA2.

**Figure 9:**
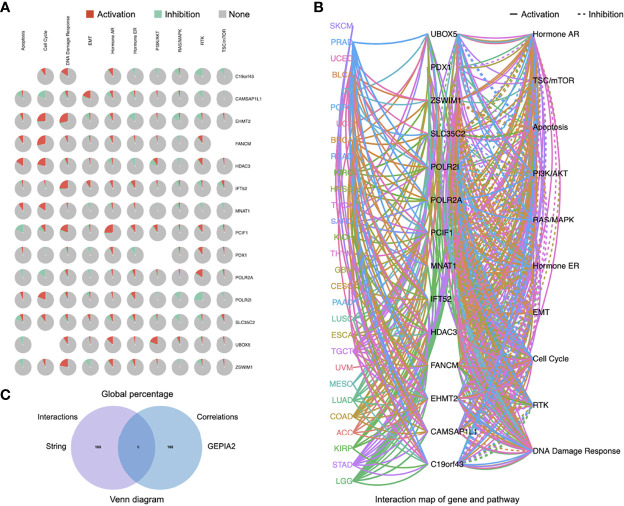
Pathway activity of eight PCIF1-correlated proteins and five PCIF1-interacted genes. **(A, B)** Global percentage and interaction map of gene and pathway of PCIF1, eight PCIF1-binding proteins (PDX1, MNAT1, HDAC3, POLR2A, POLR2I, CAMSAP2, C19orf43, FANCM), and top 5 correlated genes. **(C)** Venn diagram showed there was no overlap between PCIF1-interacted proteins and PCIF1-correlated genes.

A few progresses have been made in 2019 revealing the role of PCIF1 role as the only known m6Am methyltransferase, but still blank to cancer so far. Thus, we carried out a series of enrichment analysis of PCIF1 to explore its oncogenic role *via* STRING, GEPIA2, and GSCALite (21). Meanwhile, we gained a list of top 100 genes that have correlations with PCIF1 *via* the module of “similar gene detection” of GEPIA2 (**Supplementary Table S1**). Thirty-four proteins had an interaction with PCIF1 in *Homo sapiens* based on the STRING database; eight interacted proteins were identified by experiments, namely, PDX1, MNAT1, HDAC3, POLR2A, POLR2I, CAMSAP2, C19of43, and FANCM (**Figures 8A, B** and **Supplementary Table S2**). Pearson’s test displayed correlation of PCIF1 expression and top 5 correlated genes including ZSWIM1 (*R* = 0.55, *p* < 0.01), UBOX5 (*R* = 0.5, *p* < 0.01), SLC35C2 (*R* = 0.5, *p* < 0.01), IFT52 (*R* = 0.48, *p* < 0.01), and EHMT2 (*R* = 0.47, *p* < 0.01) (**Figure 8C**). We also obtained global percentage of PCIF1, the abovementioned eight PCIF1-binding proteins (PDX1, MNAT1, HDAC3, POLR2A, POLR2I, CAMSAP2, C19orf43, FANCM), and top 5 correlated genes (ZSWIM1, UBOX5, SLC35C2, IFT52, EHMT2) of TCGA tumors, as well as interaction map of gene and pathway (**Figures 9A, B**). PCIF1 had an activation on DNA damage response and hormone androgen receptor (AR), while an inhibition on apoptosis (**Figure 9A**). However, the Venn diagram suggested that there was no overlap between the interacted and correlated proteins (**Figure 9**).

**Figure 10:**
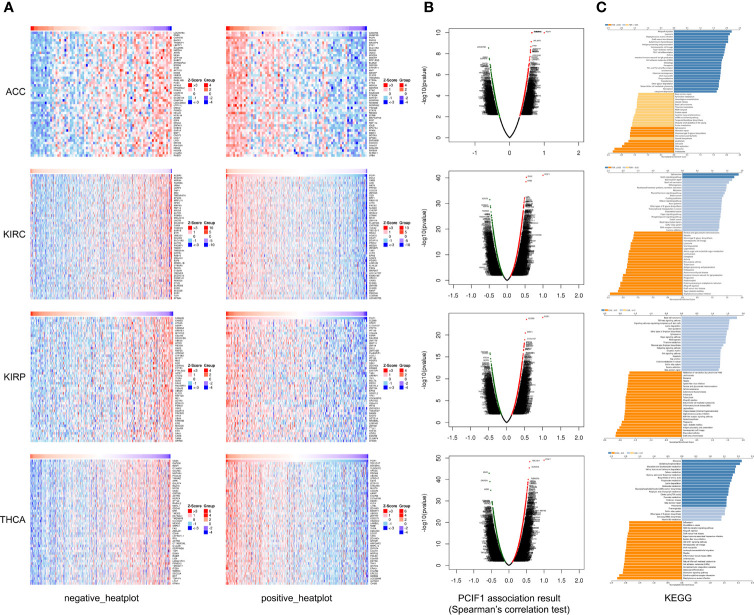
Heatplot **(A)**, Spearman’s test **(B)**, and KEGG **(C)** analyses of PCIF1-binding proteins in ACC, KIRC, KIRP, and THCA.

Furthermore, we obtained heatplots of negative or positive differentially expressed genes and Spearman’s correlation test of PCIF1-binding proteins in ACC, KIRC, KIRP, and TYHA (**Figures 10A, B**). These four tumors were those with statistical difference in normal and tumor tissues and OS and RFS as mentioned before. We defined absolute statistic value (*r* value): strong correlation for 0.7–1.0, moderate correlation for 0.4–0.7, weak correlation for 0.2–0.4, and extreme weak correlation for 0.0–0.2 (25). A positive *r* value indicated a positive correlation and vice versa. We found that in ACC, PCIF1 had a positive moderate correlation with PHF20 (*r* = 0.649), C20orf43 (*r* = 0.640), and CHMP4B (*r* = 0.638), while a negative moderate correlation with LOC90784 (*r* = −0.608), DNM1 (*r* = −0.586), and COPS7B (*r* = −0.581). In KIRC, PCIF1 had a positive moderate correlation with DVL2 (*r* = 0.536), XAB2 (*r* = 0.526), and UNK (*r* = 0.486), while a negative moderate correlation with ACER3 (*r* = −0.481), SEC24A (*r* = −0.463), and HSPA4 (*r* = −0.459) (*p* < 0.05). In KIRP, PCIF1 had a positive moderate correlation with CLDN3 (*r* = 0.549), SPEF1 (*r* = 0.516), and C17orf107 (*r* = 0.499), while a negative moderate correlation with FAM40B (*r* = −0.460), CHAC2 (*r* = −0.456), ATG4A (*r* = −0.442) (*p* < 0.05). In TYHA, PCIF1 had a positive moderate correlation with TBC1D17 (*r* = 0.594), BCKDHA (*r* = 0.579), and TUBGCP2 (*r* = 0.549), while a negative moderate correlation with UBA6 (*r* = −0.565), CAPZA1 (*r* = −0.543), and BZW1 (*r* = −0.518) (*p* < 0.05). KEGG pathway analysis revealed that PCIF1 was positively correlated with pathways associated with ferroptosis, drug metabolism, necroptosis in ACC, spliceosome, Notch signaling pathway in KIRC, ribosome, and metabolism (e.g., carbon, glycine, serine, threonine) in TYHA while negatively correlated with pathways associated with ribosome, DNA replication, cell cycle and mismatch repair in ACC, protein export, ferroptosis, proteasome in KIRC, natural killer cell-mediated cytotoxicity, antigen processing and presentation in KIRP, TNF, JAK-STAT, and NOD-like receptor signaling pathway in TYHA. However, the interaction mechanisms required more investigations.

## Discussion

Input of “PCIF1” on PubMed (https://pubmed.ncbi.nlm.nih.gov/?term=PCIF1) was quite limited, especially in cancer. Our study first analyzed PCIF1 expression and its correlation. It was shown that PCIF1 RNA expressions in most tumors was higher than that in normal tissues with significant differences but had a low specificity in tissue, blood cell lineage, each cell type, and each cell lines. Relier et al. have shown that the expression of PCIF1 and FTO was lower in metastatic cell lines, which may partially explain comparative expression in different cell lines (14). PCIF1 protein expression was different in clear cell RCC, LUAD, and UCEC. The expression of PCIF1 was correlated with OS or DFS of ACC, KIRC, KIRP, MESO, and THCA, suggesting its potential application as prognostic biomarker in the abovementioned tumors.

Uncovering oncogenic or immunogenic role of PCIF1 may be of great beneficial for cancer therapy, especially for immunotherapy or targeted therapy. Thus, we provided a primary investigation of the function of PCIF1 in tumor and immune infiltration, accompanied by limitations of this study which can only be solved by experiments. First, we had found that phosphorylation of PCIF1 at different sites resulted in differential expression of protein. However, the role of PCIF1 phosphorylation remained unclear. Secondly, we carried out a set of immune analysis to show the correlation of PCIF1 expression and TMB, MSI, immune infiltrations, and ICG expressions. These results suggested that PCIF1 might be involved in the process of immune infiltration to some extent, providing theoretic basis for the tumor immune research of PCIF1.

Taking KIRC as an example, previous studies have shown that KIRC could be classified into C3 (inflammatory) immune subtype, characterized by an elevation of helper T cell 1 (Th1) and Th17 infiltration, lower proliferation capacity, and lower level of aneuploidy and somatic CNA (23). Our bioinformatics results demonstrated that PCIF1 expression was positively correlated with OS and DFS of KIRC (**Figure 2**, *p* < 0.05) and negatively correlated with KIRC Th1 infiltration (**Supplementary Figure S2**, *p* < 0.001). Furthermore, the study of Ko et al. has shown that ZSWIM1, the top correlated genes of PCIF1, was selectively downregulated in Th1 and could be considered a biomarker for activation or differentiation of CD4+ Th cells (26). These findings suggested a potential participation of PCIF1 in regulating Th1-mediated immune modulatory effect in KIRC, while requiring experimental verifications.

Actually, though the studies of PCIF1 were quite limited, the past 4 years did witnessed great progress in this area. From 2018 to 2019, four critical studies have suggested PCIF1 as m6Am methyltransferase of mRNA and uncovered its role in promotion of mRNA stability, expression, and translation (3–7). So far, there were only two studies that demonstrated the role of PCIF1 in cancer. Zhang et al. identified circ-ATAD1/miR-140-3p/YY1/PCIF1 signaling pathway in the acceleration of gastric cancer cell progression (27). The study by Relier et al. showed that PCIF1 silence reduced colonosphere formation and m6Am/m6A ratio and caused resistance to chemotherapy in CRC1 colorectal cancer cell line (14). It remained obscure whether the participation of PCIF1 in oncogenesis and cancer development is dependent on m6Am modification. The mechanism of the oncogenic and immunogenic role of PCIF1 was a complicated puzzle. Our study tended to portray that the interaction, correlation network, and potential pathway activity were obtained from the TCGA databases with diverse formats like Pearson’s test, Spearman’s test, and KEGG analysis, hoping to guide the way for future mechanisms and functional studies of PCIF1 in cancer. However, more experimental studies are required to verify these bioinformatics results.

## Data Availability Statement

The original contributions presented in the study are included in the article/**Supplementary Material**. Further inquiries can be directed to the corresponding authors.

## Author Contributions

W-LJ and X-PW conceived and edited the paper. M-ZJ and Y-GZ did the analysis. M-ZJ wrote the paper. All authors contributed to the article and approved the submitted version.

## Funding

X-PW is supported by the National Natural Science Foundation of China (Nos. 81874103 and 81930064).

## Conflict of Interest

The authors declare that the research was conducted in the absence of any commercial or financial relationships that could be construed as a potential conflict of interest.

## Publisher’s Note

All claims expressed in this article are solely those of the authors and do not necessarily represent those of their affiliated organizations, or those of the publisher, the editors and the reviewers. Any product that may be evaluated in this article, or claim that may be made by its manufacturer, is not guaranteed or endorsed by the publisher.

## Supplementary Material

Supplementary Figure 1 Correlation between PCIF1 and overall, disease-free, progression-free, and disease-specific survival in 33 TCGA tumors.

Supplementary Figure 2 Correlation between PCIF1 and immune infiltration based on CIBERSORT, EPIC, MCP-Counter, quanTIseq, and xCell tool.

Supplementary Figure 3 Correlation between PCIF1 and immune infiltration in ACC and KIRP (*p* > 0.05).
